# The Effects of Electrical Stimulation Program on Navicular Height, Balance, and Fear of Falling in Community-Dwelling Elderly

**DOI:** 10.3390/ijerph18179351

**Published:** 2021-09-04

**Authors:** Juntip Namsawang, Pornpimol Muanjai, Nongnuch Luangpon, Sirirat Kiatkulanusorn

**Affiliations:** 1 Department of Physical Therapy, Allied Health Sciences Faculty, Burapha University, Chonburi 20131, Thailand; pornpimolm@buu.ac.th (P.M.); nongnuchl@go.buu.ac.th (N.L.); Siriratk@go.buu.ac.th (S.K.); 2 Exercise and Nutrition Innovation and Sciences Research Unit, Burapha University, Chonburi 20131, Thailand

**Keywords:** electrical stimulation, navicular, balance, fear of falling, elderly

## Abstract

Introduction: Intrinsic foot muscle weakness is a crucial cause of balance deficit in the elderly, which leads to a limited range of motion from the fear of falling and subsequently decreases the quality of life. Muscle strengthening via transcutaneous electrical stimulation (TENS) is an effective intervention; however, its effects on elderly people have rarely been reported. This study was conducted to investigate the effects of TENS on navicular height, balance, and fear of falling. Method: In this study, forty-eight participants aged 65–75 years were included and were randomly divided into two groups: the TENS and control groups. Before and after 4 weeks of training, navicular height, balance, and fear of falling were measured. Result: After 4 weeks of training, navicular height significantly increased in both groups (*p* < 0.05); however, the increase was higher in the TENS group (*p* = 0.035). The TENS group had a better improvement in balance in all four directions—front, back, left, and right (*p* < 0.05). However, postural balance improvements in the control group were observed in three directions only—front, back, and left (*p* < 0.05)—without any significant difference between the two groups. Furthermore, the TENS group decreased the scale of fear of falling after 4 weeks of training (*p* = 0.039). Conclusion: In summary, the results of this study can be used as part of the muscle strengthening via ES for decreasing the risk of falls or fear of falling in the elderly.

## 1. Introduction

People are gradually aging worldwide, and the degeneration process associated with age causes several health problems, especially an increase in the risk of falls by approximately 30% for older adults over 65 years and by approximately 40% for individuals over 70 years [1]. Falls are a health-related condition in this age and can cause many severe injuries and disabilities, which could lead to death. Once they have had any fall experience, elderly individuals usually suffer fear of falling, and this occurs linearly with age. Specifically, elderly individuals who have had fall experiences have 29–92% of fear of falling, and those who have never had fall experiences have 12–65% of fear of falling [2]. The fear of falling can lead to an increase in movement impairments, such as walking, balance, and transferring disabilities, by 56%, which subsequently affects the quality of life [3].

Poor balance is a fundamental factor of fear of falling. It has been demonstrated that the fear of falling was positively related to the balance ability in community-dwelling elderly individuals [4]. Additionally, Thiamwong and Suwanno (2017) [2] found that poor balance was a crucial factor for fear of falling in this population. Plantar foot intrinsic muscle strength clinically declines with age, and the weakness of this muscle further affects balance equilibrium [5], increasing the risk of falls by four times [6]. Specifically, the abductor hallucis (AbdH) muscle is the target muscle, among the plantar foot intrinsic foot muscles, for strength and balance training and is indeed degenerated by age-related reduced muscle mass, which directly decreases muscle strength by approximately 24–40% compared with adolescents [7]. Additionally, a study observed a navicular drop due to decreased AbdH muscle strength [8]. The magnitude of navicular drop is related to intrinsic foot muscles, where lower intrinsic foot muscle strength clinically presents with a greater navicular drop, and vice versa. The intrinsic foot muscles weakness found that this factor was the primary cause of flatfoot because the intrinsic foot muscles play a crucial role in dynamically supporting the medial longitudinal arch (MLA), reducing foot pronation as detected by the magnutude of navicular drop [9,10,11], and maintaining the balance [12]. Recent studies introduced the concept of foot core which described the important function of the plantar intrinsic foot muscles in supporting MLA. These muscles are located within the foot and act as the local stabilizers function. Therefore, these muscles are the prime dynamic stability of MLA [13,14]. This has been confirmed by Mulligan and Cook (2013) [8,11], who found an electromyographic impairment of the plantar foot intrinsic muscles leading to a decrease in the navicular height and shape of the medial longitudinal arch.

Nowadays, strengthening of the AbdH muscle can be performed in many ways; however, neuromuscular electrical stimulation (NMES) is an especially effective method for directly stimulating the targeted muscle during training compared with traditional techniques, such as the short foot exercise [15]. Symmetrical biphasic squared pulses directly generate impulses and sends them to the nerve supply of the targeted muscle for better contraction [16]. The usage of transcutaneous electrical nerve stimulation (TENS) is widespread clinically shown to reduce pain and improve function in different painful conditions and generally being one of the primary tools for managing pain [17]. The timing of TENS application can be used for 30 min long without any adverse effects in vulnerable subjects [18]. Despite pain management, TENS application is clinically recommended to aim for strengthening muscle because afferent inputs evoked by TENS enable the reaching of both sensory and motor cortices [19]. The work of Jung et al. [20] supported that lower-extremity muscle strength and postural sway improvements were substantially found following TENS use for spasticity treatment after sit-to-stand training compared with placebo. This is well supported by the previous study having beneficial effects on spasticity, strength, and mobility after the TENS procedure. Brincks and Nielsen (2012) [21] also revealed increased plantar flexor strength in patients with stroke after robotic gait training and which led to the improvement in the gait speed and subsequently improved postural balance.

Considering the anatomical structure of the AbdH muscle, the short foot exercise is inappropriate since this muscle is small; hence, the expected results may be small. A study showed that NMES generated by high-voltage pulsed current can significantly increase AbdH muscle strength after 4 weeks of training in young adults [15]. Nevertheless, the effects of home-used TENS on navicular height, balance, and fear of falling in the elderly has not been investigated yet; thus, this study was conducted to investigate the effects of TENS on navicular height, balance, and fear of falling in community-dwelling elderly individuals. The hypothesis in this study was that TENS induced navicular height, balance, and decreased fear of falling more than the short foot exercise alone. 

## 2. Materials and Methods

### 2.1. Study Design

**Figure 1:**
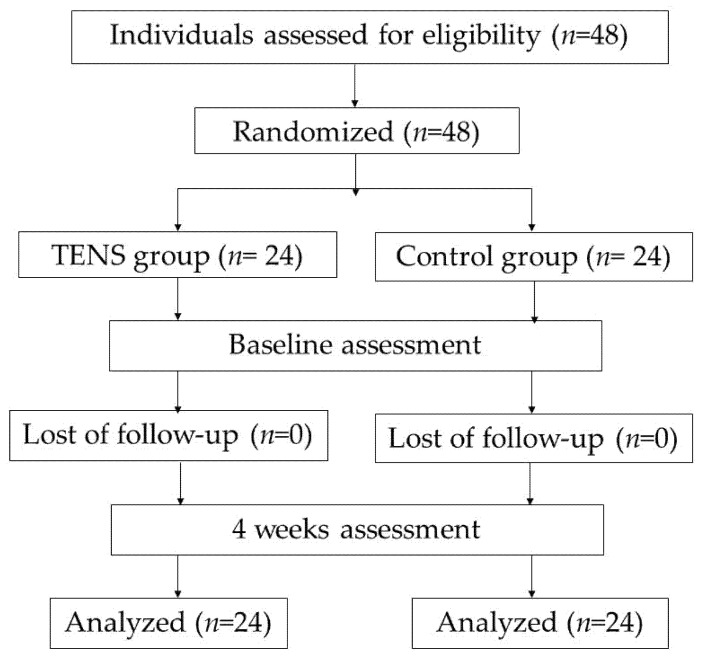
Flowchart of diagram.

This study adopted a single-blind randomized clinical trial study design. Data collection was performed from July 2020 to January 2021 in Seansook community, Muang, Chonburi. The study was approved by the Ethics Committee of Burapha University (registration number 272/2562). Block randomization was performed, and the participants were divided into two groups: 4 weeks’ TENS group and exercise control (CON) group. Before and after 4 weeks of intervention, the navicular drop test (NDT), balance, and fear of falling were measured (Figure 1). One day before the study, the agreement to the study protocol, methods, and measurements was ensured.

### 2.2. Participants

Forty-eight elderly volunteers of both sexes were included in the study. There were 3 males (age, 70.8 ± 2.9 years; weight, 70.3 ± 18.3 kg; height, 170 ± 12.0 cm for TENS group and age, 70.0 ± 2.2 years; weight, 64.8 ± 9.3 kg; height, 171.3 ± 5.8 cm for CON group) and 21 females (age, 68.2 ± 3.1years; weight, 56.0 ± 7.6 kg; height, 156.9 ± 5.9 cm for TENS group and age, 67.4 ± 5.1 years; weight, 56.9 ± 8.6 kg; height, 156.8 ± 5.9 cm for CON group) for each group. They could communicate well and walk independently without gait; aids were included in the study. Those who had any foot pain, recent lower limb fractures, or neurological deficits in the past 6 months, those wearing an implanted pacemaker, and those with an open foot wound were excluded. After the researchers informed all participants of all needed information, the participants provided written informed consent. 

### 2.3. Intervention

**Figure 2:**
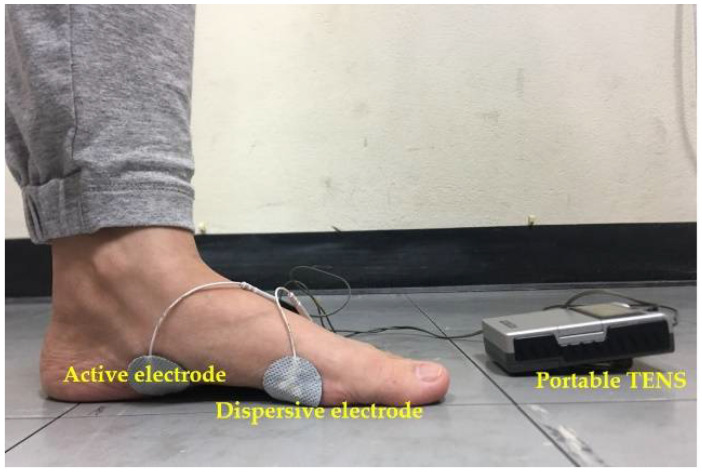
Adhesive electrode placements and portable TENS during the TENS procedure.

The TENS group received TENS pulse on the AbdH using a bipolar technique where active electrodes (32 mm, PG479, FIAB Spa, Florence, Italy) are placed on the motor point and dispersive electrodes (32 mm, PG479, FIAB Spa, Florence, Italy) are placed on the head of the first metatarsal bone [15] as shown in Figure 2. The transcutaneous electrical nerve stimulation (TENS) is a type of electrical stimulation used in this study (EV-806 TENS/EMS, Comfy Slim, Everyway medical instruments, New Taipei City, Taiwan), which generated a pulse duration of 200 ms with a frequency of 100 Hz [22]. When the electrodes were in place, the participants were asked to stand and take an equal weight distribution on both feet; then, the intensity of the impulse was slightly increased until the targeted maximum contraction was observed without any pain or discomfort present for 15 min. The procedure was repeated twice with a 2-min rest, 3 days per week for 4 weeks.

The CON group was first provided the instructions for the short foot exercise in the standing position. The short foot exercise was performed by attempting to pull the head of the metatarsal bones toward the calcaneus without flexing the toes or lifting the forefoot and heel from the ground and holding for 5 s. The exercise was repeated 15 times with 2-min rest for one set and again for another set for 30 contractions, 3 days per week for 4 weeks. The exercises in this study were home-based, which were noted in a booklet and followed up on every week by telephone.

### 2.4. Outcome Measures

The NDT was used to indicate navicular height changes in response to the interventions. Navicular height is defined as the height distance from the navicular tuberosity to the floor level, measured using a digital vernier caliper (570 series; Mitutoyo Co., Osaka, Japan) while the feet are bearing and not bearing weight. The reliability of the measurement was high (0.98) [23], and all measures were tested using the right foot. The NDT was first measured in the sitting position (non-weight-bearing) with the hip and knee flexed 90° and the feet aligned in the neutral position, and the navicular height distance was measured. Then, the navicular height distance was indicated again with the participants standing while bearing weight equally between feet, with the knee straight without any foot contractions. The three measurements of each position were noted for further analysis, and finally, the difference in the average values between positions was reported.

A force platform biofeedback system (Biometric Ltd., Newport, UK) was used to measure balance ability, where the participants first stood on the platform surrounded by a safety frame with their hands on their waist while taking weight equally between the feet. Then, the participants were instructed to lean toward four directions: forward, backward, to the right, and to the left, regardless of falls or grasping of the safety frame. Each position was held for 5 s. The procedure was repeated thrice. The maximum peak weight was noted for further analysis.

The fear of falling was assessed using the Falls Efficacy Scale International (FES-I) questionnaire in the Thai language (Cronbach’s alpha = 0.95) [2,24,25]. The Thai FES-I comprised 16 ability-related questions, including physical and psychosocial performance, using a 4-point Likert-type scale, with “1” indicating no fear at all and “4” indicating the maximum fear of falling. A total score of 16–21 points represented no fear of falling, a total score of 22–27 points indicated mild to moderate level of fear of falling, and a total score of 28–64 points represented the highest level of fear of falling [2]. 

### 2.5. Statistical Analysis

The sample size was calculated using power analysis using G*Power, version 3.1.9.2, with a significance level of 0.05, power of 90%, and effect size of 0.54. The mean and standard deviations of the navicular drop test (NDT) were used for the calculation (5.8 ± 2.1 vs. 9.8 ± 3.7 mm) [26]. The sample size totaled 36 with a 30% drop-out rate, and the total number finally was 47. Then, 24 of each group were the sample size of this study.

Data were represented as mean ± standard deviation, and the Shapiro–Wilk test was used for assessing the normality of data distribution. The physical characteristics of the participants were examined using the independent t-test and chi-square test, whereas two-way mixed analysis of variance was used to compare the changes in the NDT, balance, and FES-I in response to exercise and ES intervention. The p-values of less than 0.05 were used to denote statistical significance.

## 3. Results

The compliance of the study was excellent, and no adverse effects were observed during and after the interventions and training. The physical characteristics of both groups are shown in the participants section, and no significant differences of sex, age, body weight, height, and body mass index were observed between the two groups (*p* > 0.05).

The navicular height measured using the NDT was lower after 4 weeks of training in both groups (F(1,23) = 42.24; *p* < 0.001; ES = 0.48) (Table 1), and the interaction effect was found (F(1,23) = 8.44; *p* = 0.006; ES = 0.16). After the independent t-test was performed, the TENS group had a significantly lower NDT score than the CON group (−1.43 mm (confidence interval (CI): −2.74 to −0.11 mm); *p* = 0.035).

Postural static balance in the TENS group significantly increased in all perturbation directions (*p* < 0.001) (Table 1) whereas, except for the right direction, the CON group had a higher peak weight following the exercise control for 4 weeks (*p* < 0.001). No significant difference was observed between the two groups.

The fear of falling assessed using the FES-I was significantly lower after 4 weeks of TENS intervention (F(1,23) = 26.15; *p* < 0.001; ES = 0.36), whereas no change in the FES-I score was observed in the CON group. The interaction effect was found (F(1,23) = 8.42; *p* = 0.006; ES = 0.16) as shown in Table 1. The mean difference in FES-I scale was −2.83 points (CI: −5.52 to −0.15 points; *p* = 0.039) between the ES and CON groups after 4 weeks of training and intervention.

## 4. Discussion

This study showed that the navicular height, balance, and fear of falling improved after 4 weeks of TENS intervention, whereas the short foot exercise resulted in higher navicular height and balance ability. The TENS group had a higher navicular height and a lower fear of falling than the CON group. The clinical relevance of using TENS is that TENS impulse is speculated to enhance the strength of the plantar foot intrinsic muscles, as supported by Jung et al. (2017) [27]. A study investigated the strengthening effect of using TENS and found an increase in the strength of the plantar foot flexor muscles in elderly individuals. Another study by Okamura et al. (2018) [28] reported that TENS delayed the timing of the navicular height drop greater than the control treatment during gait analysis.

Additionally, another study revealed an increase in muscle mass by 6.6% following electrical muscle stimulation application, which was investigated using dual-energy X-ray absorptiometry [29]. The explanation of this change can be due to the neural adaptations stimulated by the generating of direct impulse to nerves inside the targeted muscle, subsequently leading to twitch contractions [27]. Moreover, electrical impulses likely reverse the physiological recruitment pattern of motor units where fast twitches become faster than slow units. Large-diameter axons in fast twitches are more likely stimulated by the electrical impulse [30]; thus, the force generated by the fast twitches during NMES is higher than that during the voluntary short foot exercise in the regular recruitment pattern [31]. Furthermore, another mechanism could be explained by the fact that motivation influences strength gain. After NMES application, all muscle fibers were stimulated to contract, and the summation of muscle force production will reach the maximum. However, voluntary muscle contraction cannot recruit all motor fibers since it always has a force deficit. The amount of force deficit depends on the individual’s motivation or voluntary activation. When it has been compared, voluntary contraction exercise has a significantly higher force deficit by 60–70% than faradic-induced electrical muscle stimulation, where the force deficit is only minimal at 10% [32]. 

The impovement of navicular drop being indirect intrinsic foot muscle weakness seen in this study could be explained that TENS application causes elicited muscle contractions and also allows for the activation of a greater proportion of type II muscle fibers compared with volitional exercise at specific intensity [17]. Kang et al. (2013) [33] also claimed that when TENS application for calf muscles was effective in improving balance in terms of increases joint position sense and strength for healthy adults, which concurs with the results of the present study and the previous study who investigated many neurological conditions [34]. 

Postural balance ability improvement was observed following 4 weeks of TENS and exercise control interventions; however, the peak weight of one direction for the CON group was unchanged. We speculated that balance ability increased due to the increased force production of the AbdH muscle, which is consistent with the findings of a previous study. They have reported that toe flexor muscle strengthening could increase either static or dynamic balance ability in elderly individuals [35]. Thus, electrical muscle stimulation is a useful intervention for increasing muscle mass and maintaining the balance function in older adults with dementia [29]. Both interventions were performed in the standing position; thus, the proprioception of the ankle and foot would be stimulated and trained, which improved the balance equilibrium ability. Proprioception plays an important role in balance equilibrium awareness where sensing proprioceptors are located inside the skin, muscle, ligament, tendon, and joint; these proprioceptors work together with visual and vestibular centers. When proprioceptors are stimulated, they send sensory input to the central nervous system; the sensory input by proprioceptors is normally stimulated at the afferent motor pathways in the sole of the foot, via the posterior white column of the spinal cord. Therefore, there would be an improvement in balance ability following the use of NMES on the foot. The result of balance improvement in the present study may be due to increased somatosensory input with muscle fatigue-impaired proprioception to the central nervous system (CNS), enhancing standing balance control after TENS application [22]. Additionally, Ferlinc et al. (2019) [36] suggested that proprioceptors are also impaired as age increases, which leads to abnormalities in joint biomechanics and the neuromuscular control of the lower limbs, resulting in impaired balance and a higher possibility of falls. One possible mechanism of the increased balance ability following rectangular-wave pulsed current and exercise control intervention would be due to an increase in the strength of the AbdH muscle in response to training. Another study found that the dynamics of postural balance after rectangular-wave pulsed current were significantly improved in the elderly. It could be due to improved musculotendinous stiffness after rectangular-wave pulsed current. Additionally, rectangular-wave pulsed current acts as extrinsic factor to produce muscle force tension. Thus, rectangular-wave pulsed current could promote both sensory input through a higher sensibility of muscle spindles and the transmission of force to the extrinsic factor to adjust the posture depending on the motor output [37].

Fear of falling score clinically improved following 4 weeks of TENS compared with the CON group, possibly due to an increase in the strength of intrinsic foot muscles in the elderly, similar to the findings of a previous study [38]. The study of Hashimoto and Sakuraba (2014) [39] confirmed that strengthening the AbdH muscle results in better standing and walking performance. Moreover, the peak weight is substantially a strong factor of fear of falling improvement, which enhances self-confidence and the ability to perform various activities. A recent study demonstrated the negative association between a history of falls and the amount of muscle mass and the positive correlation between Berg balance scale and FES-I scores and a history of falls. This could explain the association between intrinsic foot muscle hypertrophy induced by NMES indirectly measured by the NDT and decreased fear of falling found in this study [40]. Interestingly, the CON group did not have significant changes in fear of falling scores, although the strength of intrinsic foot muscles and balance ability clinically improved. These changes remain unclear for this group; however, this might be due to the amount of adaptations that insufficiently influenced the psychosocial response in the fear of falling score.

This study has some limitations. First, in this study, the training period was short. A longer period of training should be implemented to better understand the long-term results. Second, dynamic balance variables should be investigated more, such as the Timed Up and Go test. Lastly, in this study, healthy elderly individuals were investigated. These individuals might not notice the changes, as elderly individuals usually have the flatfoot deformity, a condition where intrinsic foot muscles are weak due to repetitive load bearing during walking and standing. 

## 5. Conclusions

In summary, strengthening induced by TENS could be of interest for the navicular height, balance, and fear of falling improvements compared with exercise control. These benefits may enhance navicular height and balance due to muscle strengthening and decrease the fear of falling. The results of this study suggest that portable TENS can be easily used at home and are useful effective, equipment for the early treatment/rehabilitation in terms of muscle reeducation training for muscle strengthening as a biofeedback, especially in patients with small AbdH muscles.

## Figures and Tables

**Table 1:** Mean and SDs of the NDT, balance, and FES-I before and after 4 weeks of TENS and CON interventions.

Observe Parameter	Mean ± SD				*p*-Value, ES		
	Control		TENS		Within Group Effect	Between Group Effect	Inter-Action Effect
	Pre-Test	Post-Test	Pre-Test	Post-Test			
NDT (mm)	6.60 ± 2.24	5.95 ± 2.21	6.21 ± 2.48	4.53 ± 2.33	*p* < 0.001 * ES = 0.48	*p* = 0.167 ES = 0.04	*p* = 0.006 * ES = 0.16
Peak weight: front (kg)	55.46 ± 17.20	61.73 ± 13.81	53.00 ± 18.40	66.28 ± 14.39	*p* < 0.001 * ES = 0.36	*p* = 0.806 ES < 0.01	*p* = 0.077 ES = 0.07
Peak weight: back (kg)	51.57 ± 17.67	59.13 ± 11.35	53.28 ± 16.26	64.82 ± 8.82	*p* < 0.001 * ES = 0.357	*p* = 0.305 ES = 0.02	*p* = 0.298 ES = 0.02
Peak weight: Rt. (kg)	62.42 ± 13.96	66.48 ± 12.65	53.23 ± 16.43	68.82 ± 10.85	*p* < 0.001 * ES = 0.34	*p* = 0.315 ES = 0.02	*p* = 0.007 * ES = 0.15
Peak weight: Lt. (kg)	61.59 ± 15.70	69.65 ± 13.74	57.14 ± 15.51	73.61 ± 14.02	*p* < 0.001 * ES = 0.41	*p* = 0.946 ES < 0.01	*p* = 0.06 ES = 0.08
FES-I	25.96 ± 6.42	24.63 ± 4.41	26.63 ± 6.99	21.79 ± 4.82	*p* < 0.001 * ES = 0.36	*p* = 0.488 ES = 0.01	*p* = 0.006 * ES = 0.16

TENS, transcutaneous electrical nerve stimulation; NDT, navicular drop test; FES-I, Falls Efficacy Scale; International; ES, effect size; * significant change *p* < 0.05.

## Data Availability

The datasets used and analyzed during this study are available from the corresponding author on reasonable request.
